# The neural substrates of natural reading: a comparison of normal and nonword text using eyetracking and fMRI

**DOI:** 10.3389/fnhum.2014.01024

**Published:** 2014-12-23

**Authors:** Wonil Choi, Rutvik H. Desai, John M. Henderson

**Affiliations:** Department of Psychology, Institute for Mind and Brain, University of South CarolinaColumbia, SC, USA

**Keywords:** reading, eye movements, fMRI, pseudo-reading, attention

## Abstract

Most previous studies investigating the neural correlates of reading have presented text using serial visual presentation (SVP), which may not fully reflect the underlying processes of natural reading. In the present study, eye movements and BOLD data were collected while subjects either read normal paragraphs naturally or moved their eyes through “paragraphs” of pseudo-text (pronounceable pseudowords or consonant letter strings) in two pseudo-reading conditions. Eye movement data established that subjects were reading and scanning the stimuli normally. A conjunction fMRI analysis across natural- and pseudo-reading showed that a common eye-movement network including frontal eye fields (FEF), supplementary eye fields (SEF), and intraparietal sulci was activated, consistent with previous studies using simpler eye movement tasks. In addition, natural reading versus pseudo-reading showed different patterns of brain activation: normal reading produced activation in a well-established language network that included superior temporal gyrus/sulcus, middle temporal gyrus (MTG), angular gyrus (AG), inferior frontal gyrus, and middle frontal gyrus, whereas pseudo-reading produced activation in an attentional network that included anterior/posterior cingulate and parietal cortex. These results are consistent with results found in previous single-saccade eye movement tasks and SVP reading studies, suggesting that component processes of eye-movement control and language processing observed in past fMRI research generalize to natural reading. The results also suggest that combining eyetracking and fMRI is a suitable method for investigating the component processes of natural reading in fMRI research.

## Introduction

Understanding the neural architecture of reading is one of the central issues in cognitive neuroscience (Reichle et al., 2011). Although a variety of neural aspects of reading have been understood via functional neuroimaging, most of these findings have been obtained from paradigms in which single words are presented to readers with a secondary meta-linguistic task for each word, such as lexical decision, semantic categorization, and covert or overt naming (for a comprehensive review, see Price, 2012). Even in those cases involving sentence or paragraph reading rather than single-word reading, the words have typically been presented one at a time in serial visual presentation (SVP, e.g., Martin-Loeches et al., 2008; Fedorenko et al., 2011; Pallier et al., 2011), with participants often asked to do a secondary task such as probe matching.

In contrast to single-word and SVP reading, during natural reading the eyes move through text in a series of rapid movements (saccades) and brief static periods (fixations), with mean fixation durations of 200–250 ms and mean saccade lengths of 7–9 characters (for reviews, see Rayner, 1998, 2009). The pattern of eye movements during reading is substantially correlated with linguistic factors, implying that readers' eye movements during reading reflect online cognitive processes (Rayner, 1998, 2009; Henderson, 2013). It has previously been shown that both behavioral and imaging data related to sentence processing differ for SVP and whole-sentence reading (Lee and Newman, 2010). The importance of eye movements in natural reading was recently highlighted by an eyetracking study reported by Schotter et al. (2014), showing that sentence comprehension is negatively affected when regressive eye movements are not available during reading.

In the majority of fMRI studies that have presented an entire sentence or passage at once (e.g., Ferstl and von Cramon, 2001; Bohrn et al., 2013; Altmann et al., 2014; Hsu et al., 2015), eye movements have not been monitored, so it has not been possible to investigate questions concerning the integration of language comprehension and eye movement control during natural reading. In a recent study, Hillen et al. (2013) examined how neural activation is elicited by eye movements in text by asking subjects to move their eyes through sentences in an fMRI study. The authors compared fMRI activation for normal sentences, scrambled sentences, nonword sentences, and pseudo-text made up of Landolt rings (circle-like shapes). Hillen et al. found activation of a common gaze network across these conditions that included bilateral frontal eye fields (FEF), supplementary eye field (SEF), and right intraparietal sulcus (rIPS), the same areas reported in other fMRI studies using simple eye movement tasks like the pro- and anti-saccade tasks (Paus et al., 1993; Sweeney et al., 1996; Everling and Munoz, 2000; Ford et al., 2005; Ettinger et al., 2008, for a recent review, see Jamadar et al., 2013). Although the Hillen et al. study suggests that sequential reading-like tasks activate the same eye movement network that has been observed in simpler saccadic oculomotor tasks, subjects were not actually engaged in natural reading. Instead, subjects were asked to detect characters that looked like a “left-opened o” (Landolt C) that were embedded in the real and pseudoword texts. These instructions might cause subjects to use relatively different (e.g., more controlled) scanning strategies than those used in natural reading. In a related study, Richlan et al. (2014) compared reading materials (words or pronounceable nonwords) to non-reading materials (lines or Hebrew characters) using a fixation-related fMRI analysis method. However, in that study, the items were presented in arrays and did not comprise connected text, so the task was quite different than natural reading.

Given the importance of eye movements in natural reading, it is surprising that the neurocognitive basis of natural reading in which readers actively move their eyes through units of text (e.g., sentences or paragraphs) is largely unknown. In the current study, we pursued three goals. The first two were related to the nature of the eye movement control network during reading. First, we investigated the general characteristics of the eye movement network when participants sequentially move their eyes through text and text-like stimuli. In their study, Hillen et al. (2013) reported evidence for a gaze control network that was common across reading and pseudo-reading conditions, though subjects were engaged in a search task. In the current study, we examined the nature of the eye-movement network common to natural reading and sequential pseudo-reading tasks that did not include a search or other secondary task. Our second goal was to investigate how activation in the eye movement network differs in natural reading and pseudo-reading by directly comparing these conditions. Our third goal was to investigate the language processing network in normal reading. Previous studies have shown that SVP reading produces a distinct pattern of neural activation compared with pseudo-reading involving nonword strings or false fonts (Noppeney and Price, 2004; Fedorenko et al., 2011, 2012; Hillen et al., 2013). It is not currently known whether this pattern generalizes to natural reading in which subjects actively control their eye movements and therefore control the timing and order of text encoding and analysis.

In sum, the present study was designed to investigate the nature of the networks activated when participants read naturally via eye movements, compared to pseudo-reading controls. We simultaneously recorded eye movements and BOLD activity while subjects read passages of text or moved their eyes through similarly arranged pseudo-text made up of pronounceable pseudowords or consonant strings. Because we were interested in natural reading, in the reading condition subjects simply read naturally with no secondary task. To facilitate natural reading, we presented full paragraphs rather than sentences, and the paragraphs were connected across trials in coherent passages.

## Methods

### Subjects

Thirty-three subjects (12 male) participated in this study. Two of them did not finish the experiment. Therefore, 31 participants' data were included in the analysis. They were all right-handed native speakers of English, aged 18–35 years (Mean Age: 21.48). Thirty subjects were students from the University of South Carolina and three were recruited from the community in Columbia, South Carolina. All subjects gave informed consent and were screened for MRI safety, following the ethics protocol approved by the Institutional Review Board of the University of South Carolina. All subjects reported normal or corrected-to-normal vision and were given $10 per hour for participation in the study.

### Materials

The experiment consisted of three conditions: Normal Text (NT), Pseudoword Text (PW), and Consonant String Text (CS). In the NT condition, 22 paragraphs were selected from two sources, *The Emperor's New Clothes* by Hans Christian Andersen (11 paragraphs), and a Nelson-Denny Practice Test (11 paragraphs). Paragraphs consisted of 49 to 66 words. In the PW condition, 22 paragraphs were created with pseudowords that were generated from the ARC Nonword Database (available at http://www.psy.uwa.edu.au/MRCDataBase/uwa_mrc.htm). The pseudowords were in accordance with the phonotactic rules of English so that they were pronounceable. The CS condition included 22 paragraphs with consonant-string nonwords that were created using randomly chosen consonants. Text was presented in Courier New font (monospaced) with 4.3 characters subtending 1° of visual angle. All nonword stimuli were matched to the words used in the NT condition with respect to the number of lines, the number of words, word length, and the position of punctuation.

### Apparatus

Stimuli were presented using an Avotec Silent Vision 6011 projector in its native resolution (1024 × 768) and a refresh rate of 60 Hz. Eye-movements were monitored via a SR Research Eyelink 1000 long-range MRI eyetracker with a sampling rate of 1000 Hz. Viewing was binocular and eye-movements were recorded from the right eye.

### Procedure

In the scanner, a thirteen-point calibration procedure was administrated before each of the two functional runs to correctly map eye position to screen coordinates. Eye movements were recorded throughout the runs to ensure that natural reading eye-movements were executed during in the NT condition and that scanning eye movements were executed in the PW and CS conditions.

Each functional run consisted of 11 normal text paragraphs (the NT condition), 11 pseudoword paragraphs (the PW condition), and 11 consonant string paragraphs (the CS condition), as well as 11 filler trials containing pictures not relevant to the current study. Each trial was presented for 12 s preceded by a fixation cross for 6 s. Within each run, normal texts, pseudo texts and filler trials were presented in a random order for each participant. Participants therefore saw 22 trials in each condition over the two runs. Each functional run lasted about 14 min. Participants were asked to read paragraphs silently as if they were reading a novel when a text paragraph was presented, and to move their eyes “as if they were reading” in the PW and CS conditions.

### MRI data acquisition

MR data were collected on a Siemens Medical Systems 3T Trio. A 3D T1-weighted “MPRAGE” RF-spoiled rapid flash scan in the sagittal plane, and a T2/PD-weighted multi-slice axial 2D dual Fast Turbo spin-echo scan in the axial plane was used. The multi-echo whole brain T1 scans had 1 mm isotropic voxel size and sufficient field of view to cover from the top of the head to the neck with the following protocol parameters: *TR* = 2530 ms, *TE*1 = 1.74 ms, *TE*2 = 3.6 ms, *TE*3 = 5.46 ms, *TE*4 = 7.32 ms, flip angle = 7°. All functional runs were acquired using gradient echo, echo-planar images with the following protocol parameters: *TR* = 1850 ms, *TE* = 30 ms, flip angle = 75°. Volumes consisted of thirty-four 3 mm slices with transversal orientation. Each volume covered the whole brain with FOV = 208 mm and 64 × 64 matrix, resulting in 3.3 × 3.3 × 3 mm voxel size.

### MRI analysis

The AFNI software package (Cox, 1996) was used for image analysis. Within-subject analysis involved slice timing correction, spatial co-registration (Cox and Jesmanowicz, 1999) and registration of functional images to the anatomy (Saad et al., 2009). Voxel-wise multiple linear regression was performed with the program 3dREMLfit, using reference functions representing each condition convolved with a standard hemodynamic response function. Reference functions representing the six motion parameters were included as covariates of no interest. In addition, the signal extracted from CSF and white matter was also included as noise covariates of no interest. General linear tests were conducted to obtain contrasts between conditions of interest.

The individual statistical maps and the anatomical scans were projected into standard stereotaxic space (Talairach and Tournoux, 1988) and smoothed with a Gaussian filter of 5 mm FWHM. In a random effects analysis, group maps were created by comparing activations against a constant value of 0. The group maps were thresholded at voxelwise *p* < 0.01 and corrected for multiple comparisons by removing clusters with below-threshold size to achieve a mapwise corrected *p* < 0.05. Using the 3dClustSim program with 10000 iterations, the cluster threshold was determined through Monte Carlo simulations that estimate the chance probability of spatially contiguous voxels exceeding the voxelwise p threshold, i.e., of false positive noise clusters. The smoothness of the data was estimated with the AFNI program 3dFWHMx using regression residuals as input. The analysis was restricted to a mask that excluded areas outside the brain, as well as deep white matter areas and the ventricles.

## Result

### Eye-movements results

Table 1 shows basic eye-movement data for each condition. Data during track losses were eliminated and fixations meeting the following criteria were excluded from this analysis: A fixation made before or after a blink and fixation durations less than 50 ms or greater than 1500 ms. In total, 12.5% of fixations (11.3% for the NT condition, 13.5% for the PW condition, and 13% for the CS condition) were excluded from analysis. As seen in Table 1, mean fixation duration was statistically different across the three conditions, *F*_(2, 62)_ = 56.67, *p* < 0.001, in that the NT condition had shorter fixation durations than the average of the PW and CS conditions, *F*_(1, 31)_ = 62.58, *p* < 0.001. There was no difference in mean fixation duration between the PW and the CS condition, *F*_(1, 31)_ = 0.004, *p* = 0.948. The pattern of results for mean fixation duration was also found in the first-pass reading time measures [first fixation duration (FFD), single fixation duration (SFD), and gaze duration (GD)], [*F*_(2, 62)_ = 42.92, *p* < 0.0001 for FFD; *F*_(2, 62)_ = 39.17, *p* < 0.0001 for SFD; *F*_(2, 62)_ = 33.79, *p* < 0.0001 for GD], with those for the NT condition shorter relative to the average of the PW and the CS conditions, [*F*_(1, 31)_ = 49.3, *p* < 0.0001 for FFD, *F*_(1, 31)_ = 47.16, *p* < 0.0001 for SFD, *F*_(1, 31)_ = 39.72, *p* < 0.0001 for GD], and no difference between the PW and the CS conditions, *F*s < 1, ns. Saccadic amplitude also differed across the three conditions, *F*_(2, 62)_ = 19.91, *p* < 0.001, with the NT condition producing greater saccadic amplitude than the average of the two nonword text conditions, *F*_(1, 31)_ = 19.92, *p* < 0.001, and the PW condition producing greater saccadic amplitude compared with the CS condition, *F*_(1, 31)_ = 19.63, *p* < 0.0001. The proportion of regressions (RegProp) also differed across the three conditions, *F*_(2, 62)_ = 6.92, *p* < 0.005, with the NT condition producing more regressions than the average of the PW and the CS conditions, *F*_(1, 31)_ = 8.01, *p* < 0.01, and a marginal difference between the PW and CS conditions, *F*_(1, 31)_ = 4.0, *p* = 0.054. This general pattern of results in eye movements was similar to that reported in an analogous fMRI study (Henderson et al., 2014) and those obtained outside the scanner comparing natural and false-font texts (e.g., Henderson and Luke, 2012, 2014; Luke and Henderson, 2013).

**Table 1:** **Summary eye movement data**.

		**NT**	**PW**	**CS**
Fixation duration (ms)	Mean	218	261	261
	Standard deviation	25	45	41
Saccade amplitude (deg)	Mean	2.86°	2.51°	2.42°
	Standard deviation	0.47°	0.72°	0.68°
FFD (ms)	Mean	222	267	269
	Standard deviation	28	54	49
SFD (ms)	Mean	223	271	274
	Standard deviation	29	58	52
GZD (ms)	Mean	252	340	338
	Standard deviation	39	111	88
RegProp (%)	Mean	10.3	9.4	8.8
	Standard deviation	0.04	0.03	0.28

*Mean fixation duration and saccade amplitude for each condition as a function of condition averaged over subjects. NT, Normal Text; PW, Pseudoword Text; CS, Consonant String Text; FFD, First Fixation Duration; SFD, Single Fixation Duration; GZD, Gaze Duration; RegProp, Proportion of inter-word regression*.

### fMRI results

The fMRI results are displayed on inflated brain surfaces using caret5 (Van Essen et al., 2001). The complete lists of activated areas per contrast are provided in Tables 2–**6**.

**Table 2:** **Talairach coordinates, volume of the cluster (μl), maximum z-score, and the label of anatomical structure for the normal text (NT) condition > fixation analysis, L, left hemisphere; R, right hemisphere**.

**Volume**	**Max**	***x***	***y***	***z***	**Anatomical structures**
248373	7.601	4	−70	−18	R/L Cerebellum, R/L Cuneus, R/L Lingual Gyrus
	7.492	−52	7	−9	L Superior Temporal Gyrus
	7.279	22	−25	0	R Ventral Diencephalon
	7.177	−19	−91	−3	L Occipital Pole
	6.864	−25	−19	−3	L Superior Colliculus, L Ventral Diencephalon, L Thalamus, L Putamen, L Caudate
	6.66	−58	−31	2	L Superior Temporal Gyrus/Sulcus, L Middle Temporal Gyrus, L Fusiform Gyrus
	6.392	−40	−1	50	L Middle Frontal Gyrus, L Precentral Gyrus, Lateral Frontal Eye Field
	5.604	10	4	14	R Superior Colliculus, R Caudate, R Thalamus, R Putamen
	5.424	28	−94	0	R Occipital Pole
	4.47	−40	40	−6	L Pars Orbitalis, L Pars Triangularis
25866	6.655	49	−31	2	R Superior Temporal Gyrus/Sulcus, R Middle Temporal Gyrus
15363	6.397	−1	1	59	Supplementary Eye Field, L/R Superior Frontal Gyrus
	5.426	−4	40	50	L/R Superior Frontal Gyrus
10503	5.355	52	28	5	R Parstriangularis, R Middle Frontal Gyrus, R Precentral Sulcus
1377	4.627	−25	−55	44	L Intraprietal Sulcus
1215	3.844	31	−67	26	R Intraprietal Sulcus
1188	3.629	−4	−28	56	L/R paracentral Gyrus/Sulcus

#### Normal text—fixation

**Figure 1:**
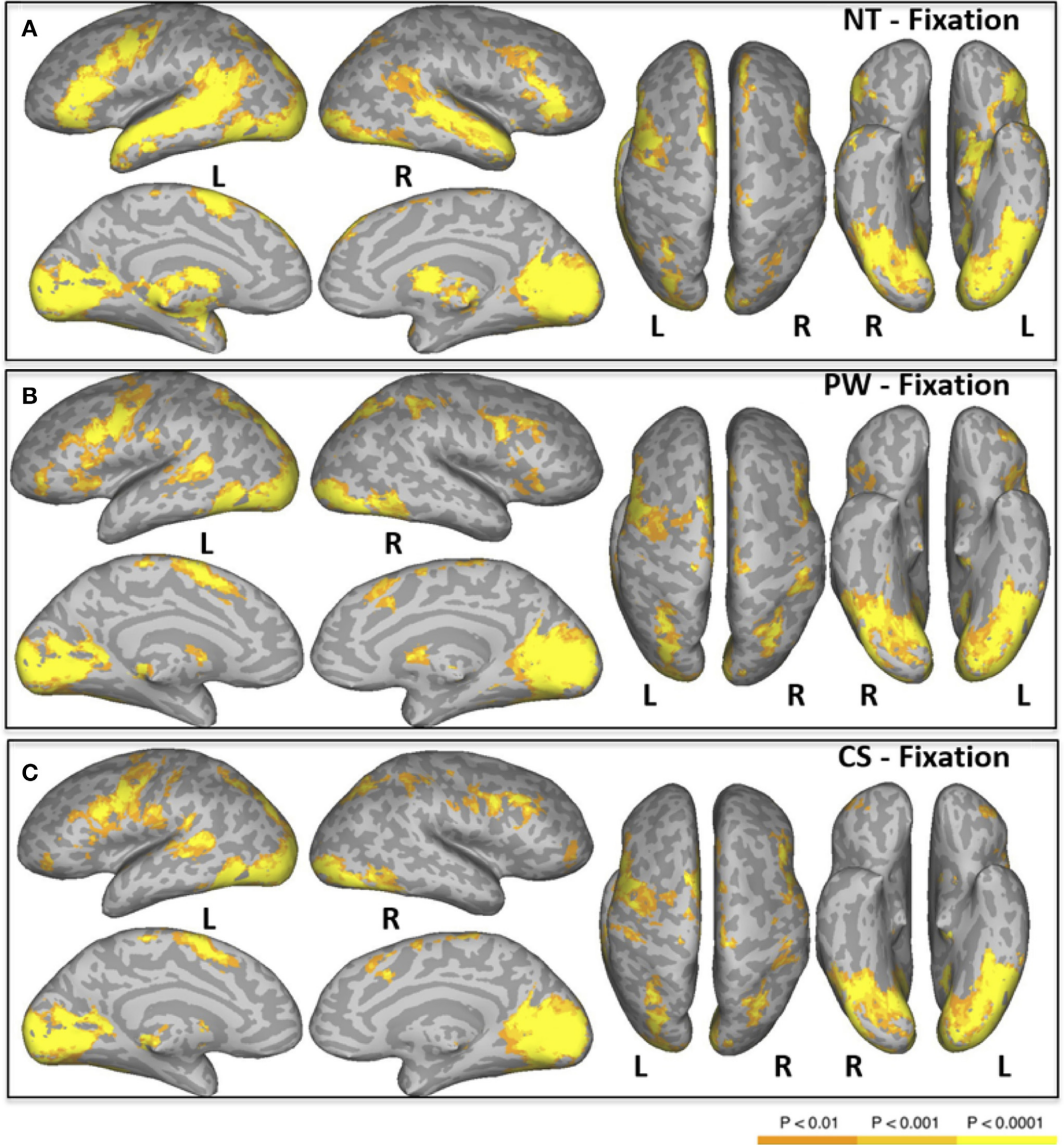
**Areas of significant activation in a whole-brain analysis of natural reading of normal text [NT condition, (A)], and “reading” pseudowords [PW condition, (B)] and consonant strings [CS condition, (C)]**. L, Left Hemisphere; R, Right Hemisphere.

Areas activated during natural reading of normal text are shown in Figure 1A and Table 2. These included cortical and subcortical areas associated with the eye-movement control network: bilateral FEF, SEF, bilateral IPS, bilateral superior colliculus (SC), and bilateral thalamus. Areas related to language processing were also strongly activated including bilateral middle temporal gyrus (MTG), bilateral superior temporal gyrus/sulcus (STG/STS), bilateral inferior frontal gyrus (IFG), and angular gyrus (AG). In addition, areas involved in visual processing were activated: bilateral cuneus, bilateral lingual gyrus, bilateral occipital pole, and left fusiform gyrus (FG).

#### Pseudowords—fixation

Areas activated during pseudo-reading of pseudowords are shown in Figure 1B and Table 3. Activation was seen in bilateral FEF, SEF, and IPS, as well as left STS and bilateral IFG. Visual activation was also observed in bilateral cuneus and left occipital cortex. Subcortical activation was seen in caudate, putamen, and pallidum.

**Table 3:** **Talairach coordinates, volume of the cluster (μl), maximum z-score, and the label of anatomical structure for the pseudoword text (PW) condition > fixation analysis, L, left hemisphere; R, right hemisphere**.

**Volume**	**Max**	***x***	***y***	***z***	**Anatomical structures**
121203	7.01	−37	−82	−6	L Middle Occipital Gyrus
	6.561	7	−76	5	R/L Cuneus
	5.717	31	−58	−21	R Cerebellum
	4.801	−25	−55	41	L Intraparietal Sulcus
	4.49	−13	−55	−27	L Cerebellum
24462	5.664	−43	1	35	L Precentral Sulcus, L Frontal Eye Field
	4.18	−40	40	2	L Inferior Frontal Gyrus, L Orbital Gyrus
9693	5.444	−1	1	59	Supplementary Eye Field, L/R Superior Frontal Gyrus
6534	4.784	43	1	26	R Precentral Sulcus/Gyrus, R Middle Frontal Gyrus, R Lateral Frontal Eye Field
4887	5.804	−19	−22	0	L Ventral Diencephalon, L Thalamus
4374	4.713	−49	−37	8	L Posterior Superior Temporal Sulcus
4347	4.736	28	−61	32	R Intraprietal Sulcus
3024	4.151	13	13	5	R/L Caudate, R/L Putamen, R/L Pallidum
2997	4.177	4	−19	−27	Brain-stem
2538	4.47	43	−37	41	R Postcentrral Sulcus
1998	3.741	40	22	2	R Inferior Frontal Gyrus
1026	5.484	22	−22	0	R Ventral Diencephalon, R Thalamus

#### Consonant strings—fixation

Areas activated during pseudo-reading of consonant strings are shown in Figure 1C and Table 4. Activation was seen in bilateral FEF, SEF, and IPS, left posterior STS (LpSTS), bilateral IFG, middle frontal gyrus (MFG), bilateral orbital gyrus, occipital cortex, and FG.

**Table 4:** **Talairach coordinates, volume of the cluster (μl), maximum z-score, and the label of anatomical structure for the consonant string text (CS) condition > fixation analysis, L, left hemisphere; R, right hemisphere**.

**Volume**	**Max**	***x***	***y***	***z***	**Anatomical structures**
114831	7.062	−19	−91	−6	L Occipital Pole, L Middle Occipital Gyrus
	6.656	7	−70	8	R Cuneus, R Occipital Pole
	6.291	−40	−64	−18	L Fusiform Gyrus, L Cerebellum
	5.654	34	−58	−21	R Cerebellum, R Fusiform Gyrus, R Inferior Occipital Gyrus
	4.511	−25	−70	35	L Intraparietal Sulcus, L Superior Parietal Gyrus
23409	6.428	−46	−1	44	L Precentral Gyrus, L Frontal Eye Field, L Inferior Frontal Gyrus, L Middle Frontal Gyrus
8343	4.883	40	1	29	R Precentral Sulcus/Gyrus, R Frontal Eye Field, R Middle Frontal Gyrus
5265	4.931	−46	−37	8	L Posterior Superior Temporal Sulcus
5076	5.781	−1	1	59	Supplementary Eye Field, L/R Superior Frontal Gyrus
2430	4.296	−4	−31	56	L/R Paracentral Sulcus/Gyrus
1863	3.861	49	−31	47	R Postcentral Gyrus
1863	4.491	−25	−55	44	L Intraparietal Sulcus, L Superior Parietal Gyrus
1701	3.968	−34	34	8	L Orbital Gyrus
1242	4.032	46	−22	56	R Postcentral Gyrus
1107	4.09	−10	−22	−27	Brain-stem
945	4.453	13	13	35	R Anterior Cingulate
918	3.66	43	52	0	R Orbital Gyrus, R Middle Frontal Gyrus
918	5.313	22	−22	0	R Ventral Diencephalon, R Thalamus

#### Conjunction of normal text, pseudowords, and consonant strings

**Figure 2:**
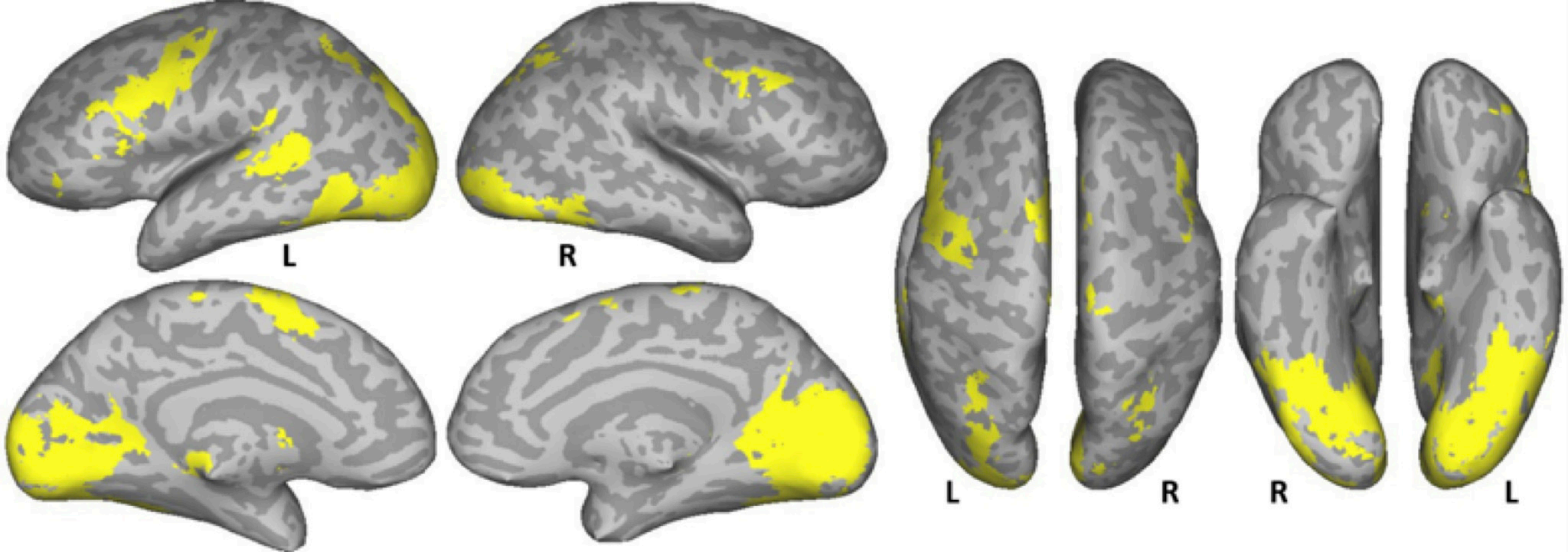
**Conjunction results for the normal text (NT), pseudowords (PW), and consonant string (CS) conditions (NT – fixation n PW− fixation n CS− fixation)**. L, Left Hemisphere; R, Right Hemisphere.

A conjunction analysis of the NT, PW, and CS conditions was conducted to examine the common eye-movement network. Activation in this contrast would also be expected for areas involved in processing character strings (e.g., orthographic and potentially phonological processing) related to the presence of alphabetic characters. Figure 2 shows the results. Activation was observed in bilateral FEF, SEF, and IPS. Activation was also observed in LpSTS, left IFG, left precentral gyrus, and left MFG (premotor area, BA6).

#### Normal text vs. average of pseudowords and consonant strings

**Figure 3:**
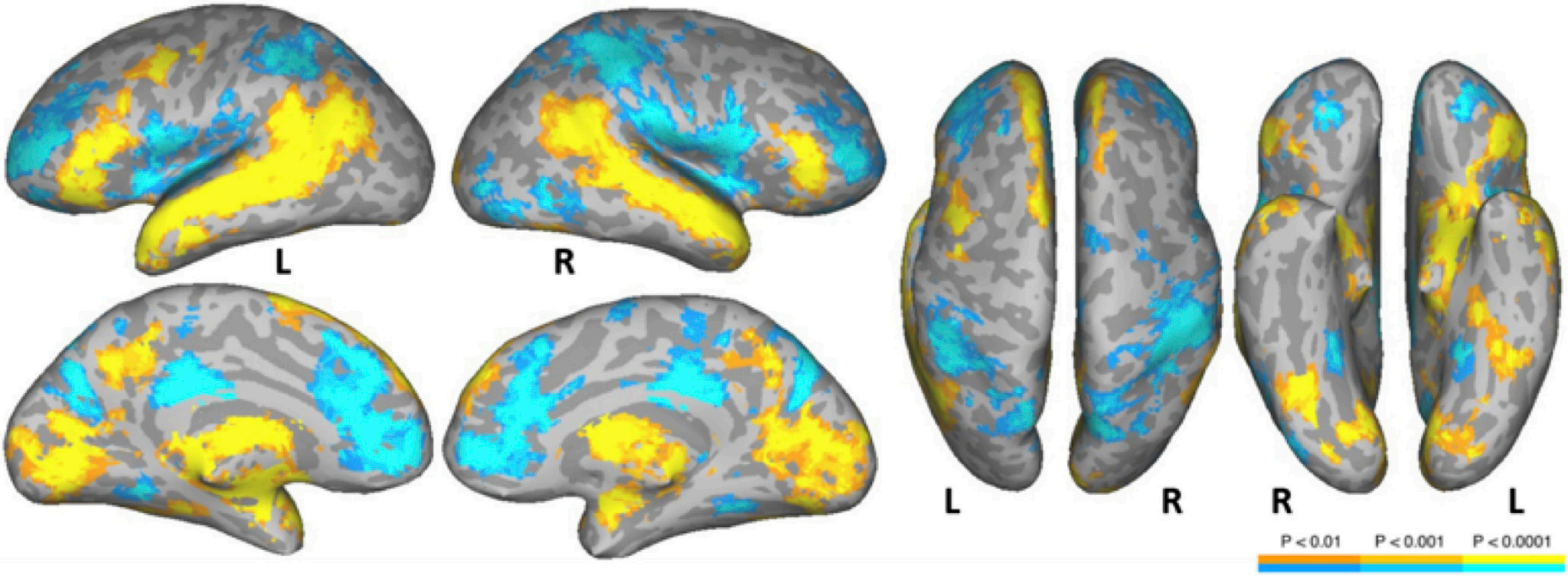
**Areas activated in a whole-brain analysis of the contrast of natural text reading (NT) versus the average of pseudowords and consonant strings (mean of PW and CS)**. Hot regions represent more activation for text whereas cool regions reflect more activation for nonwords. L, Left Hemisphere; R, Right Hemisphere.

The NT condition was compared to the two nonword conditions to examine normal reading versus pseudo-reading. The PW and CS conditions produced similar patterns of activation against fixation baseline (see Figures 1B,C), so these conditions were averaged for this comparison. Activated areas are shown in Figure 3 and Table 5. Areas producing greater activation in the NT condition were left MFG including lateral FEF, bilateral SFG including pre-SMA, bilateral STS and anterior STG, bilateral MTG, bilateral AG, bilateral IFG (pars triangularis), bilateral cuneus, and bilateral precuneus. Subcortical activation was seen in caudate, thalamus, and ventral diencephalon.

**Table 5:** **Talairach coordinates, volume of the cluster (μl), maximum z-score, and the label of anatomical structure for the normal text vs. the nonword texts analysis, L, left hemisphere; R, right hemisphere**.

**Volume**	**Max**	***x***	***y***	***z***	**Anatomical structures**
**NORMAL TEXT > NONWORD TEXTS**					
130653	7.682	−52	7	−9	L Anterior Superior Temporal Gyrus, L Middle Temporal Gyrus, L Inferior Frontal Gyrus (Pars Triangularis)/Sulcus, L Middle Frontal Gyrus
	6.669	−52	−31	2	L Posterior Superior Temporal Sulcus, L Angular Gyrus
	6.563	−7	−28	2	L/R Thalamus, L/R Superior Colliculus, L/R Ventral Diencephalon, L Fusiform Gyrus
	5.844	10	7	14	R/L Caudate, R/L Thalamus, R/L Ventral Diencephalon
66825	7.276	16	−67	−24	R Cerebellum
	5.168	−1	−70	11	L/R Cuneus, R Occipital Pole, L Cerebellum
35775	6.985	46	7	−15	R Anterior Superior Temporal Gyrus, R Middle Temporal Gyrus
	6.573	58	−37	2	R Middle Temporal Gyrus, R Angular Gyrus, R Superior Temporal Sulcus
17145	5.935	−7	43	50	L/R Superior Frontal Gyrus
	5.218	−4	10	62	L Superior Frontal Gyrus, L Pre-Supplementary Motor Area
5589	4.715	−4	−58	32	L/R Precuneus, R Subparietal Sulcus
5481	5.567	−40	−1	50	L Middle Frontal Gyrus, L Lateral Frontal Eye Field
4077	5.589	52	28	5	R Inferior Frontal Gyrus (Pars Triangularis)
**NONWORD TEXTS > NORMAL TEXT**					
51705	−5.929	40	−37	41	R Postcentral Sulcus/Gyrus, R Supramarginal Gyrus, R Superior Parietal Gyrus, R Intraparietal Sulcus
	−5.363	46	1	2	R Inferior Frontal Gyrus (Pars Opercularis), R Subcentral Gyrus/Sulcus, R Transverse Temporal Gyrus/Sulcus, R Insula
	−5.259	13	−67	41	R Superior Parietal Gyrus, R Parieto-Occipital sulcus, R Superior Occipital Gyrus
35937	−7.399	−4	31	14	L/R Anterior Cingulate Gyrus/Sulcus, L/R Superior Frontal Gyrus
	−5.436	−34	55	14	L Middle Frontal Gyrus/Sulcus
	−5.922	−40	−37	38	L Postcentral Sulcus/Gyrus, L Supramarginal Gyrus
	−5.817	−37	−22	5	L Transverse Temporal Gyrus/Sulcus, L Insula
	−5.767	−34	13	5	L Anterior Insula, L Inferior Frontal Gyrus (Pars Opercularis)
	−5.137	−19	−64	23	L Parieto-occipital sulcus
9882	−5.466	40	52	11	R Middle Frontal Gyrus/Sulcus
7047	−5.753	−1	−22	26	L/R Posterior Cingulate Gyrus
4725	−4.779	34	−79	0	R Middle Occipital Gyrus
2916	−5.069	31	−40	0	R Hippocampus
945	−3.733	−46	−58	−33	L Cerebellum
945	−4.995	25	34	−6	R Orbital Sulcus
945	−4.796	−28	−43	0	L Hippocampus
945	−3.471	7	−4	65	R Superior Frontal Gyrus
918	−3.634	25	−13	47	R Precentral Sulcus

Greater activation was produced in the average of the two nonword conditions in bilateral SFG, bilateral anterior and posterior cingulate, bilateral supramarginal gyrus, bilateral transverse temporal gyrus/sulcus, bilateral MFG/MFS, bilateral postcentral gyrus/sulcus, bilateral hippocampus, bilateral parieto-occipital sulcus, bilateral insula, bilateral IFG (pars opercularis), bilateral superior parietal lobule and IPS, right subcentral gyrus/sulcus, right superior occipital gyrus, right middle occipital gyrus, right orbital sulcus, and right precentral sulcus.

#### Pseudowords vs. consonant strings

**Figure 4:**
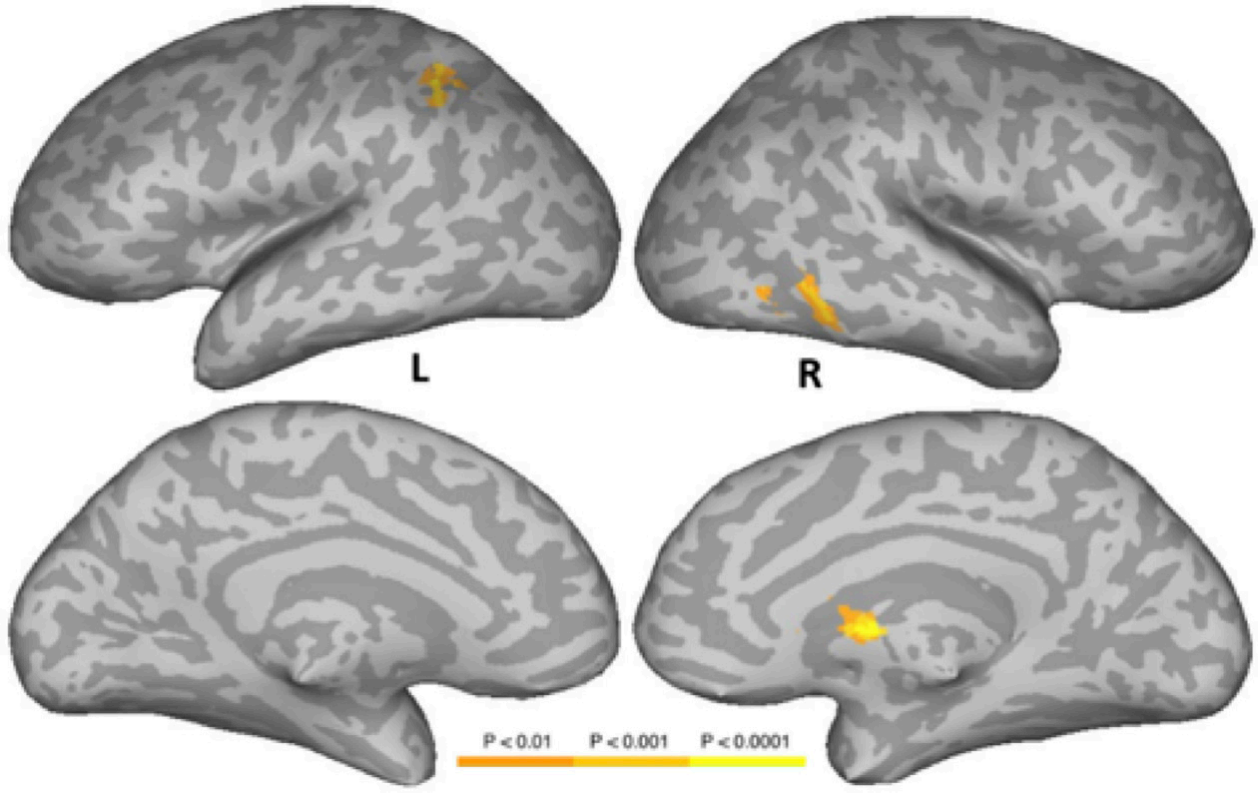
**Areas activated in a whole-brain analysis of the contrast of the pseudowords versus consonant string conditions**. Hot regions represent more activation in the pseudowords condition than in the consonant string condition. L, Left Hemisphere; R, Right Hemisphere.

Figure 4 and Table 6 show the results of a PW versus CS contrast. Activation was greater in the PW condition in left IPS, right inferior temporal gyrus (ITG), right FG, and right caudate. No regions produced more activation in the CS condition.

**Table 6:** **Talairach coordinates, volume of the cluster (μl), maximum z-score, and the label of anatomical structure for the pseudo text vs. the consonant string text analysis, L, left hemisphere; R, right hemisphere**.

**Volume**	**Max**	***x***	***y***	***z***	**Anatomical structures**
**PSEUDOWORD TEXT > CONSONANT STRING TEXT**					
1215	3.95	−34	−43	41	L Intraparietal Sulcus
1080	3.861	46	−49	−9	R Inferior Temporal Gyrus, R Fusiform Gyrus
1026	4.107	7	7	8	R Caudate
**CONSONANT STRING TEXT > PSEUDOWORD TEXT**					
None					

## Discussion

This study was designed to investigate the neural correlates of natural reading. Subjects read passages of text presented in paragraph form while both eye movements and the BOLD signal were recorded. The natural reading condition was compared to two pseudo-reading conditions in which words were replaced by either pronounceable pseudowords or consonant strings. In the latter two conditions subjects were asked to move their eyes through the text “as if they were reading” (Vitu et al., 1995; Rayner and Fischer, 1996; Nuthmann and Engbert, 2009; Reichle et al., 2010; Henderson and Luke, 2012, 2014; Luke and Henderson, 2013). Three specific questions were addressed: the nature of the common eye-movement control network in these sequential scanning tasks, the nature of the eye movement network in natural reading, and the nature of the language network in natural reading.

### Common eye movement control network

A common eye movement control network was revealed across the three conditions. As can be seen in Figure 2, bilateral FEF, bilateral IPS, and SEF were activated when sequential and complex eye movements were executed during these task. The observed areas are consistent with those that have been proposed as the core eye movement control network, as observed in single-saccade eye movement tasks (Pierrot-Deseilligny et al., 2004; McDowell et al., 2008; Müri and Nyffeler, 2008; Jamadar et al., 2013). These results are also consistent with those of Hillen et al. (2013) in which subjects searched text and pseudo-text for Landolt C targets. The results suggest that this core eye movement control network is functional in single-saccade eye-movements tasks, sequential search tasks, and in natural reading.

The activated regions reported here were very similar to those reported by Hillen et al. (2013) in their conjunction analysis across five conditions to identify the common gaze control network. However, the network reported by Hillen et al. did not include the left IPS, whereas the current results showed bilateral IPS activation. Although both Hillen et al. and the present study measured BOLD responses with sequential oculomotor paradigms rather than the SVP paradigm, Hillen et al. employed a secondary search task, which was not used in the present study. This difference between the two studies might explain why slightly different patterns of activation were observed in the eye movement control network. Note that simple eye-movement tasks usually generate activation in bilateral IPS (see Jamadar et al., 2013), implying that the IPS in both hemispheres is involved in saccade control and/or attentional processes. Hillen et al. did not discuss why activation in the left IPS was not obtained in the conjunction analysis in their paper, but did discuss the right IPS activation with respect to attentional processes (Culham et al., 2006) and planning of movements (Barthélémy and Boulinguez, 2002). Activation of the IPS in eye movement control might be associated either with saccadic movement, attentional processes, or saccade-related attentional processes. As Pierrot-Deseilligny et al. (2004) pointed out, it is relatively difficult to separate activation by saccades from activation by attentional processes, because they typically co-occur. Indeed, covert attention and eye movement control are tightly linked in reading (Henderson and Ferreira, 1990), and this link forms the basis of several current computational models of reading, (e.g., Reichle et al., 1998; Engbert et al., 2005). Further research will be needed to differentiate the neurobiology of these processes in natural reading.

### Eye movement control network for reading

The contrast analysis between normal reading and pseudo-reading showed that lateral FEF was more activated during normal reading compared to pseudo-reading. McDowell et al. (2008) proposed that lateral FEF is more associated with visually guided saccadic eye movements. In addition, Jamadar et al. (2013), in their meta-analysis, found more lateral FEF activation in pro-saccades relative to a fixation control, supporting the idea that lateral FEF is more involved in visually guided automatic eye movements. Although eye movements during natural reading are not as simple and reflexive as those in the pro-saccade task, they are highly automatized and guided by visual information from the upcoming word in the parafovea. This similarity might account for the greater activation that was observed in lateral FEF in the normal reading condition relative to the pseudo-reading conditions. At the same time, the pseudo-reading conditions showed more activation in the eye movement control network including bilateral IPS, as well as ACC, relative to the normal reading condition. These structures have been reported to be associated with both attentional processes and eye movement control (Pierrot-Deseilligny et al., 2004). Although, as indicated above, it is very difficult to functionally differentiate attentional control from eye movement control, this result may suggest that pseudo-reading requires more attentional control and/or more fine-grained eye movement coordination compared to normal reading, consistent with the idea that eye movement control is more automatized in natural reading.

### Other regions of the common network

In addition to the eye-movement network, activation across the three conditions was also observed in left posterior MFG and premotor area, left posterior IFG, and posterior STS. These regions are not commonly thought to be related to eye movement control, and could be associated with task-dependent processing. For example, premotor and posterior IFG activation could be related to covert articulation (McGuire et al., 1996; Ghosh et al., 2008; Peeva et al., 2010; Rogalsky and Hickok, 2011). In the two nonword reading conditions, subjects may have been able to pronounce the nonwords in both the PW and CS conditions, activating phonological representations and phonological working memory. Because we matched strings in length across conditions, many of the consonant strings may have been pronounceable because they were one- to three-letters long. For example, a two-letter consonant string like *sp* can be pronounced.

The conjunction analysis also showed activation of the LpSTS which has been suggested to be a region of multi-functionality (Hein and Knight, 2008; Liebenthal et al., 2014). Here, the likely role of LpSTS is also in phonological processing as part of a dorsal/posterior pathway that represents transient phonological representations and maps them to articulatory codes in premotor and posterior inferior frontal regions (Wise et al., 2001; Hickok and Poeppel, 2007; Desai et al., 2008; Obleser and Eisner, 2009). Note that the normal reading condition activated these areas (left IFG/MFG and LpSTS) to a greater extent than the pseudo-reading conditions in the contrast analysis (see Figure 3), suggesting greater and more automatic phonological processing in normal reading compared to pseudo reading.

### Linguistic and related cognitive processes

Reading paragraphs for meaning requires many levels of language representation and processing including orthographic/phonological processing, lexical access, retrieval of lexico-semantic information, syntactic computation, and semantic interpretation. The language network observed in previous studies, including STG, STS, MTG, IFG, MFG, AG, and precuneus were also more activated during normal paragraph reading compared to the pseudo-reading conditions in the present study. Regions in the lateral temporal lobe, AG, and precuneus form the core of a semantic network observed in numerous studies that typically use word or sentence stimuli (Binder et al., 2009). This network was activated strongly for natural reading of text relative to the nonword conditions in the present study, extending these past findings to natural text reading.

At the same time, the nonword conditions showed more activation in medial frontal/ACC, posterior cingulate cortex, and bilateral IPS, areas associated with attentional brain networks (for a recent review, see Petersen and Posner, 2012). The greater attentional network activation observed here for nonwords suggests that “reading” paragraphs with nonword stimuli requires substantial attentional resources, extending findings from several single-word studies that show a similar pattern (e.g., Price et al., 1996; Hagoort et al., 1999; Mechelli et al., 2000; Paulesu et al., 2000; Binder et al., 2005; Vigneau et al., 2006). In our nonword reading conditions, readers were asked to imitate normal reading patterns with eye movements, which is a relatively unpracticed task that requires encoding nonwords, inhibiting neighbor words, and coordinating eye movement control. These processes likely require more attentional resources than natural reading. Hillen et al. (2013) also reported that a condition in which text was replaced by Landolt rings showed more activation in the right inferior parietal lobule relative to the conditions that used alphabetic characters, suggesting that “Landolt reading” similarly requires more attentional resources compared to the other conditions. In addition to the neural data, the behavioral eye movement data in the current experiment support this idea in that fixation durations in nonword reading were longer than those for normal reading, indicating that more effort is necessary for nonword reading than for normal reading (for similar findings, see Henderson and Luke, 2012, 2014; Henderson et al., 2014). Another way to state this is that natural reading is highly automatized and therefore requires less attentional control than does consciously executing similar sequences of eye movements.

The text vs. nonword comparison also showed activation in bilateral IFG, both for text and for nonwords, consistent with previous single word and sentence processing studies. The IFG has a well-established role in language, including possible semantic, syntactic, phonological, articulatory, and executive functions (e.g., Hagoort, 2005; Grodzinsky and Santi, 2008; Friederici, 2009; Duncan, 2010; Rogalsky and Hickok, 2011). Regions of the anterior IFG overlapping BA 47 and 45, activated more for text, likely reflect semantic retrieval processes. The posterior IFG, activated to a greater extent for nonwords, likely reflects more effortful covet articulation and phonological processing. Additionally, as argued by Duncan (2010), posterior IFG is part of a multiple-demand network that includes posterior IFS, anterior insula/frontal operculum, pre-SMA/ACC, and IPS, and is associated with cognitive control. In the current study, nonword reading requires more effortful processing to perform complex saccadic coordination relative to normal reading because no linguistic information is provided in foveal or parafoveal vision, whereas sequential saccadic movements in normal reading can be guided by linguistic information both at the fovea and parafovea. Accordingly, readers are likely to use a less efficient control mechanism for eye movements in nonword reading relative to normal reading, as indicated by the greater activation in frontoparietal areas including PO, anterior insula, and IPS.

In summary, the present study showed that (1) activation of a core eye-movement control network was observed when participants naturally read text paragraphs or moved their eyes through nonword text, (2) differences in activation of the eye movement control network in natural reading and pseudo-reading suggest that readers use automatized saccadic coordination during natural reading whereas they require more complex attentional and control processes during pseudo-reading, and (3) normal reading produced distinct patterns of neural activation in a language-related network, extending previous findings with word and sentence stimuli. These results indicate that presenting entire paragraphs (or sentences) in fMRI during natural reading can provide important data with respect to the neural understanding of language processing and eye-movement control in reading.

### Conflict of interest statement

The authors declare that the research was conducted in the absence of any commercial or financial relationships that could be construed as a potential conflict of interest.
